# Propulsive Force Characterization of a Bio-Robotic Sea Lion Foreflipper: A Kinematic Basis for Agile Propulsion

**DOI:** 10.3390/biomimetics10120831

**Published:** 2025-12-12

**Authors:** Anthony Drago, Nicholas Marcouiller, Shraman Kadapa, Frank E. Fish, James L. Tangorra

**Affiliations:** 1 Mechanical Engineering and Mechanics, Drexel University, Philadelphia, PA 19104, USA; 2 Department of Biology, West Chester University, West Chester, PA 19393, USA

**Keywords:** biomimetics, bio-inspired robotics, California sea lion, foreflipper propulsion, hydrodynamics, propulsive forces, kinematics, force vectoring, agile propulsion

## Abstract

Unmanned underwater vehicles (UUVs) capable of agile, high-speed maneuvering in complex environments require propulsion systems that can dynamically modulate three-dimensional forces. The California sea lion (*Zalophus californianus*) provides an exceptional biological model, using its foreflippers to achieve rapid turns and powerful propulsion. However, the specific kinematic mechanisms that govern instantaneous force generation from its powerful foreflippers remain poorly quantified. This study experimentally characterizes the time-varying thrust and lift produced by a bio-robotic sea lion foreflipper to determine how flipper twist, sweep, and phase overlap modulate propulsive forces. A three-degree-of-freedom bio-robotic flipper with a simplified, low-aspect-ratio planform and single compliant hinge was tested in a circulating flow tank, executing parameterized power and paddle strokes in both isolated and combined-phase trials. The time-resolved force data reveal that the propulsive stroke functions as a tunable hybrid system. The power phase acts as a force-vectoring mechanism, where the flipper’s twist angle reorients the resultant vector: thrust is maximized in a broad, robust range peaking near 45°, while lift increases monotonically to 90°. The paddle phase operates as a flow-insensitive, geometrically driven thruster, where twist angle (0° optimal) regulates thrust by altering the presented surface area. In the full stroke, a temporal-phase overlap governs thrust augmentation, while the power-phase twist provides robust steering control. Within the tested inertial flow regime (Re ≈ 10^4^–10^5^), this control map is highly consistent with propulsion dominated by geometric momentum redirection and impulse timing, rather than circulation-based lift. These findings establish a practical, experimentally derived control map linking kinematic inputs to propulsive force vectors, providing a foundation for the design and control of agile, bio-inspired underwater vehicles.

## 1. Introduction

Operating effectively in dynamic and cluttered underwater environments requires vehicles capable of agile, three-dimensional maneuvering. Unmanned Underwater Vehicles (UUV_S_) have made advances in range and endurance, but they lack the fine, high-speed maneuverability of biological swimmers, limiting their use in complex settings like nearshore currents and riverine habitats [1,2]. Current UUVs are functionally constrained by their propulsion methods. While multi-thruster vehicles offer precision control at low speeds, they often require bulky hulls and are limited by fixed-axis thrust lines. Conversely, streamlined, propeller-driven bodies are efficient for long-range travel but lack the ability to rapidly modulate or reorient thrust for complex turns or evasive maneuvers [3]. Overcoming these limitations necessitates a propulsion system capable of generating and redirecting forces dynamically in three dimensions [4].

**Figure 1:**
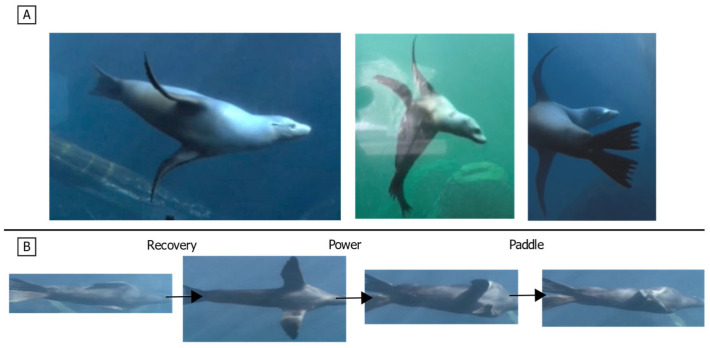
California sea lion swimming and characteristic propulsive stroke. (**A**) Sea lion maneuvering underwater using its foreflippers, hind flippers, and body for agile control. (**B**) Sequential frames from underwater video showing the three phases of the characteristic foreflipper stroke: Recovery, during which the flippers extend laterally and anteriorly away from the body; Power, where the flippers move downward and medially to generate thrust; and Paddle, as the flippers are pulled posteriorly toward the body to complete the stroke cycle and reset for the next recovery phase.

Biological swimmers demonstrate that reorientable propulsors are key to generating the dynamic, three-dimensional forces needed for agile maneuvering. A wide range of taxa exploit this principle: fish employ undulatory fin motions [5,6,7,8,9]; rays and turtles use flapping appendages for lift-based propulsion [10,11,12]; and cephalopods generate thrust through pulsed jet flows [13,14,15,16]. Among aquatic vertebrates, the California sea lion (*Zalophus californianus*) provides a highly relevant model. Sea lions use foreflippers to execute a characteristic three-phase stroke that facilitates powerful propulsion and enables exceptional agility, demonstrated by minimum turning radii as small as 0.16 body lengths and turn rates exceeding 600 deg s^−1^ [17,18,19,20,21]. While the sea lion flipper morphology and stroke kinematics are well-described, the specific influence of systematic kinematic variations on the instantaneous, time-varying magnitude and direction of the propulsive forces remains poorly quantified [Figure 1].

**Figure 2:**
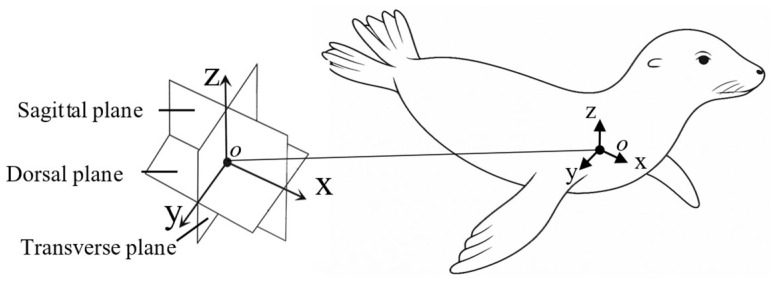
Anatomical coordinate system of the California sea lion. Body-fixed coordinate frame and associated anatomical planes used to describe foreflipper motion. The *x*-axis is oriented anteroposteriorly along the body midline toward the head, the *y*-axis extends laterally, and the *z*-axis extends dorsoventrally. These axes define the Sagittal (x − z), Transverse (y − z), and Dorsal (x − y) planes, which, respectively, separate the left and right sides, dorsal and ventral regions, and anterior and posterior portions of the body.

**Figure 3:**
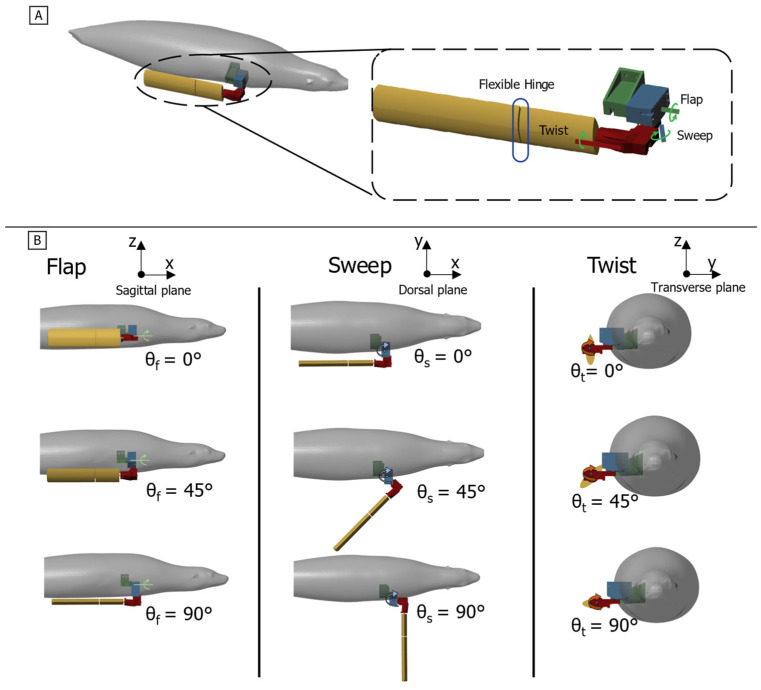
Bio-robotic foreflipper coordinate system and actuation scheme. (**A**) The bio-robotic foreflipper replicates sea lion forelimb motion through three serially arranged actuators (flap, sweep, and twist). A flexible hinge at the wrist introduces passive bending representative of the biological joint (**B**) Representative actuator motions showing the range of rotation for each axis: flap angle (θ_f_), sweep angle (θ_s_), and twist angle (θ_t_). The zero position aligns the flipper parallel to the body flank with the leading edge downward. Increasing actuator angles illustrate the independent and combined rotations that generate the full spatial envelope of sea lion foreflipper movement.

The objective of this work is to characterize the time-varying thrust and lift produced by a bio-robotic foreflipper executing the California sea lion’s characteristic propulsive stroke, with the goal of identifying the mechanisms that enable agile force modulation. This study addresses a key knowledge gap by experimentally resolving the complete, time-varying force trajectories over an entire propulsive cycle using a bio-robotic model of the foreflipper. Variations in principal stroke kinematics, including flipper rotation about its long axis (twist), forward and backward motion (sweep), and the degree of overlap between stroke phases, are systematically quantified to determine how these parameters influence the instantaneous generation and reorientation of propulsive forces [Figure 2 and Figure 3]. The results provide fundamental hydrodynamic data that are essential for the design and control of agile, bio-inspired underwater systems capable of complex three-dimensional maneuvers.

Prior investigations have established the feasibility of sea-lion-inspired propulsion, but have primarily relied on simulated, phase-averaged, or studies of the fluidics around the fore-flippers [22]. Subsequent work began to probe the propulsive mechanisms, with many studies asserting that thrust production, especially during the power phase of the stroke is mainly a lift-based phenomenon, produced by vortex-mediated mechanisms and angle-of-attack effects [23]. More recent studies have built upon this foundation, using robotic models or CFD simulations analyze phase-specific propulsion, often focusing on these hydrodynamic, lift-based interpretations [24,25]. These analyses identified vortex structures and pressure differentials consistent with lift-based effects, but they did not provide a time-resolved, systematic mapping of how specific kinematic parameters translate to measurable forces throughout a complete stroke. The present study extends this foundation by providing the first systematic, experimentally measured mapping between kinematic parameters and the resulting time-varying propulsive forces. While prior research has extensively characterized the lift-based hydrodynamics typical of high-aspect-ratio flippers, this study focuses on quantifying the geometric momentum redirection mechanisms that govern propulsion in the inertial regime.

This paper first details the design of the bio-robotic foreflipper, the experimental environment, and the kinematic parameters used to model the propulsive stroke. It then presents an analysis of the baseline force trajectories for each stroke phase. The detailed results follow, illustrating how systematic variations in key kinematics (twist, sweep, and phase overlap) modulate the instantaneous forces. These results are discussed in the context of propulsive mechanisms and bio-inspired design, followed by a conclusion that summarizes the main contributions.

## 2. Materials and Methods

### 2.1. Overview

A bio-robotic model of a California sea lion foreflipper was developed to experimentally evaluate how foreflipper kinematics affect the propulsive forces produced during a characteristic sea lion stroke. The robot consists of three main components: (1) the foreflipper model, (2) a three-degree-of-freedom (3-DOF) joint at the base, and (3) an air-bearing carriage that rigidly attaches the robot to a force measurement system above a circulating flow tank. The propulsive stroke was modeled using data collected from video footage of sea lions during natural swimming, which serves as the baseline stroke. Experiments were partitioned into three trial groups—isolated power, isolated paddle, and combined power/paddle—to evaluate the impact of the characteristic stroke and kinematic deviations on thrust and lift forces.

### 2.2. Flipper Coordinate Frame and Actuation Scheme

A body-fixed coordinate system defined the motion relative to the sea lion’s anatomy. The *x*-axis aligns along the body midline (positive toward the head), the *y*-axis extends laterally, and the *z*-axis extends dorsoventrally (positive dorsal). These axes define the Sagittal (x − z), Transverse (y − z), and Dorsal (x − y) anatomical planes [Figure 2] [17,19,20].

The robotic system employs a serial chain of three actuators (flap, sweep, and twist) to reproduce the flipper’s compound, three-dimensional motion. The flap (θ_f_), sweep (θ_s_), and twist (θ_t_) angles are defined relative to their local actuator axes [Figure 3].

The Flap actuator (θ_f_) rotates about the *x*-axis, producing motion in the Transverse plane (y − z).

The Sweep actuator (θ_s_) rotates about the *z*-axis. At its 0° position, this produces motion in the Dorsal plane (x − y).

The Twist actuator (θ_t_) rotates about the flipper’s long axis, which is parallel to the *x*-axis at the 0° home position.

The zero position (0°) for all joints places the flipper flat against the body flank. This serial configuration produces dynamic kinematic coupling: as upstream joints move, the orientation of downstream axes changes. This means the nominal association between the actuator axes and the anatomical planes becomes approximate, resulting in the non-orthogonal, compound rotations consistent with the biological foreflipper stroke.

For testing, the bio-robotic flipper was oriented as if the sea lion were swimming on its side. Thrust was defined as the force component parallel to the primary flow direction (*x*-axis), positive in the posterior direction. Lift was defined as the force component normal to the flow direction (*z*-axis), positive dorsally [Figure 2].

### 2.3. Bio-Robotic Foreflipper Model and Testing Environment

#### 2.3.1. Bio-Robotic Foreflipper Model

**Figure 4:**
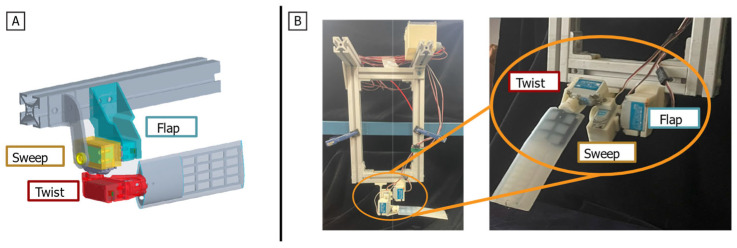
Bio-robotic Single Flipper Robot. (**A**) CAD model of the single flipper robot with the actuatable axes labeled (**B**) Full bio-robotic single flipper robot with (**Left**) support structure, control box, and actuators and (**Right**) a closer view of the flipper and actuator set-up.

**Figure 5:**
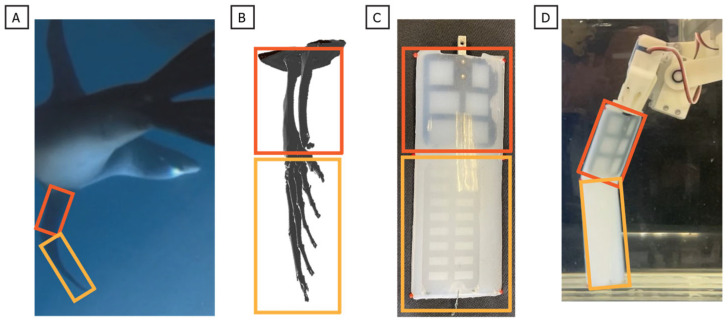
Design Of Engineered Sea Lion Flipper. (**A**) Foreflipper bending during sea lion swimming; (**B**) 3D model of sea lion foreflipper showing bone structure; (**C**) Final design of foreflipper model, highlighting the two rigid sections cast in flexible silicon; (**D**) Foreflipper model bending during actuation.

The foreflipper model was designed with a simplified, constant-chord rectangular profile to isolate the hydrodynamics of the main stroke kinematics, distinct from complex planform effects (e.g., taper and high-aspect-ratio lift) [Figure 4]. Due to test environment constraints, the model length was scaled down to 0.170 m and the chordwise width to 0.065 m. To enhance force magnitude and improve the signal-to-noise ratio, the chordwise width was increased by 35%, resulting in a modified low aspect ratio of 2.7 (compared to the biological range of 4.1 to 7.9) [19,20]. The model was fabricated with a 3D-printed base and a laser-cut acrylic tip [Figure 5].

A single compliant silicone hinge was incorporated to replicate the passive bending observed in the biological wrist. The hinge stiffness was iteratively tuned to match the curvature and deformation observed in video footage of natural sea lion swimming [Figure 5]. The compliant wrist hinge was formed using a silicone joint (Shore A hardness 30, 10 mm axial separation, 13 mm maximum thickness, 62 mm chord length). This passive element exhibited tip-to-base angle deflections ranging from 45° to 60° depending on hydrodynamic loading. This range is consistent with curvatures observed in biological flippers and emulates the flexibility of the sea lion wrist’s cartilage and connective tissue. This single-hinge design was tuned to replicate the characteristic geometric deformation observed in natural strokes, rather than the complex, distributed flexibility of the biological flipper.

The flipper base uses a three-degree-of-freedom (3-DOF) joint to replicate the compound rotations of the sea lion shoulder. The joint consists of three waterproof servomotors (WR-7701, XpertRC, WA, USA) connected in series: flap, sweep, and twist. The servomotors were operated in standard position-control mode driven by Pulse Width Modulation (PWM) signals via a microcontroller. Given that the torque capacity of the actuators significantly exceeded the hydrodynamic loads generated during testing, the system effectively enforced the prescribed kinematic trajectories without significant deviation. Consequently, the implementation of complex external feedback control loops, such as custom PID tuning, was not required for this hydrodynamic characterization. This serial arrangement enables coordinated motion across all three axes, as detailed in Section 2.1.

#### 2.3.2. Experimental Testing Environment

**Figure 6:**
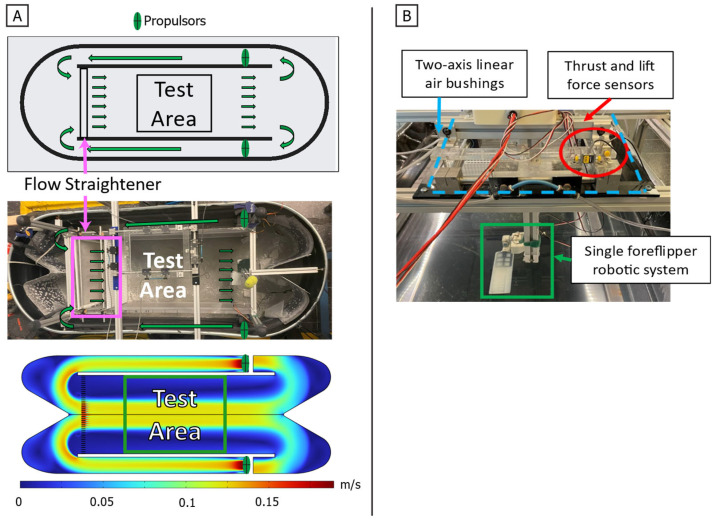
Experimental test environment. (**A**) Schematic, photograph, and computational fluid dynamics (CFD) simulation of the circulating flow tank used for experiments. Water is driven by dual propulsors and passes through three flow straighteners before entering the central test area. The CFD velocity map confirms uniform flow distribution and stable velocity profiles within the test region. (**B**) Experimental apparatus showing the suspended measurement system, including two-axis linear air bushings for low-friction translation, thrust and lift force sensors for load measurement, and the single bio-robotic foreflipper mounted within the test area. Green arrows represent flow direction.

The foreflipper assembly is mounted on an air-bearing carriage that provides low-friction support for precise force measurements. The carriage is rigidly attached to the force measurement system, which is suspended over a circulating flow tank [Figure 6]. Because the bio-robotic flipper was rotated 90° on the carriage for testing, the two measured axes directly correspond to the propulsive forces. The system incorporates two single-axis force transducers (LSB200, FUTEK, Irvine, CA, USA) connected to signal conditioners (IA100, FUTEK, Irvine, CA, USA) to measure thrust (force parallel to flow) and lift (force normal to flow).

The experimental setup used a circulating flow tank with a central test section measuring 0.45 m in width, 0.30 m in depth, and 0.50 m in length [Figure 6]. Flow conditioning was achieved through a mesh screen that reduced large-scale turbulence, followed by three flow straighteners spaced 5 cm apart to promote laminar inflow. The tank generated a spatially uniform and repeatable wash of water, maintaining controlled velocities between 0 m s^−1^ and 0.1 m s^−1^. This configuration produced stable flow conditions with moderate turbulence, representative of biologically relevant near-field swimming environments. Spatial flow uniformity within the test section was verified through pointwise velocity measurements across the width and complementary CFD analysis of the flow regime. The flow field was found to be uniform within the central test region, with a gradual velocity decrease near the side boundaries; the robotic flipper was positioned entirely within the uniform flow region during all experiments [Figure 6].

Given the geometric scale, the experimental Reynolds numbers (Re) ranged from 3.3 × 10^4^ to 6.6 × 10^4^. Crucially, this range remains well within the inertial flow regime (Re ≫ 10^3^), where propulsion is dominated by inertial and pressure forces rather than viscous effects. This provides a dynamically appropriate model for examining the hydrodynamic behavior of a momentum-driven propulsor. Neither Strouhal number nor Angle of Attack (α) is reported. Strouhal number is inapplicable as the stroke is impulsive and nonperiodic, rather than a steady oscillation and is commonly not reported when discussing the California sea lion’s propulsive foreflipper stroke [17,18]. A single representative angle of attack (α) was not reported or measured because determining the instantaneous relative flow vector in a three-degree-of-freedom (3-DoF) robotic experiment is exceptionally complex. The angle of attack depends on the orientation of the flipper’s leading edge relative to the local flow velocity, which varies as a function of both the oncoming flow and the flipper’s own powerful self-induced motion.

### 2.4. Kinematics of the Propulsive Stroke

#### Stroke Phase Description

**Figure 7:**
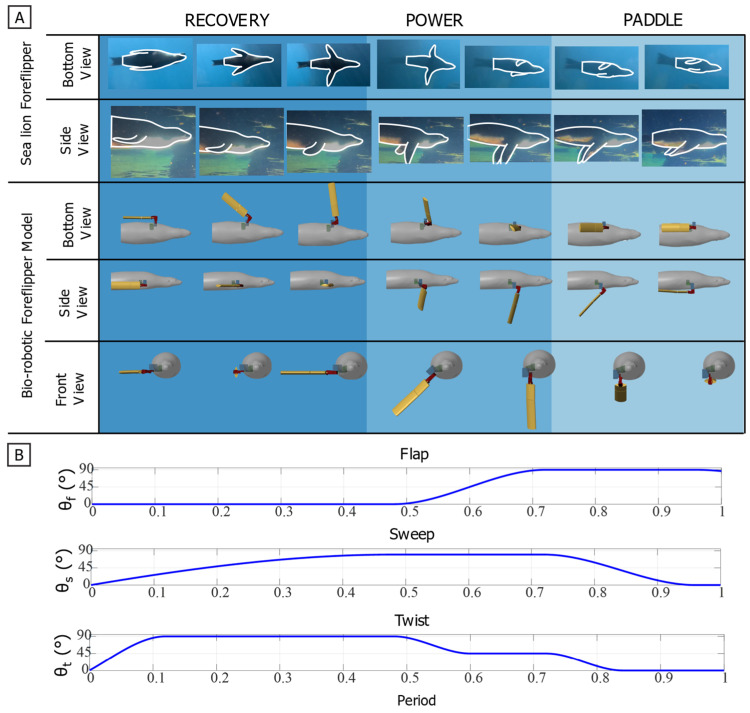
Characteristic foreflipper stroke of the California sea lion and corresponding robotic implementation. (**A**) Sequential views of the biological and bio-robotic foreflipper illustrating the three primary phases of the characteristic propulsive stroke: Recovery, where the flipper moves upward and outward to a laterally extended position minimizing drag; Power, characterized by a strong downward and rearward motion that generates thrust; and Paddle, where the flipper moves ventrally toward the body while maintaining lift before returning to the streamlined position. The bio-robotic foreflipper reproduces these coordinated motions through flap (θ_f_), sweep (θ_s_), and twist (θ_t_). (**B**) Parameterized joint trajectories for flap, sweep, and twist angles across one full stroke cycle, modeled using a piecewise cubic Hermite interpolating polynomial (PCHIP) fit to biological kinematic data. Together, these actuator profiles recreate the continuous, nonplanar motion and characteristic feathering of the sea lion’s foreflipper during propulsion.

**Figure 8:**
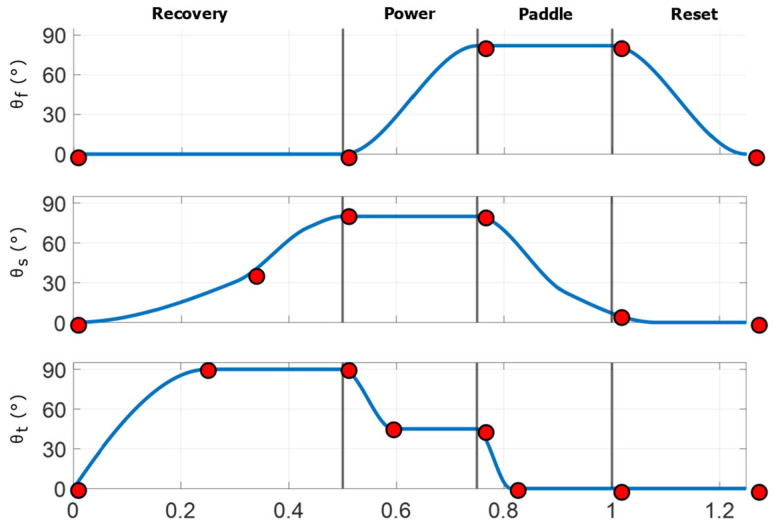
PCHIP spline representation of foreflipper kinematics. Parameterized flap (θ_f_), sweep (θ_s_), and twist (θ_t_) angles over one complete stroke cycle, divided into the Recovery, Power, Paddle, and Reset phases. Red circles indicate control points defining the piecewise cubic Hermite interpolating polynomial (PCHIP) splines used to generate smooth, shape-preserving joint trajectories without overshoot. These control points can be adjusted to modify amplitude, timing, and phase relationships between the three motions, enabling controlled variation of stroke kinematics in experiments while faithfully reproducing the characteristic sea lion stroke observed in the animal.

The foreflipper stroke of the California sea lion can be divided into three primary phases: recovery, power, and paddle [17,19,20]. A complete stroke cycle lasts approximately one second, with the power and paddle phases comprising about 60% of the cycle and the recovery phase the remaining 40%. During the recovery phase, the flipper moves upward and outward (abduction) to a laterally extended position, minimizing drag while preparing for propulsion. The power phase follows, characterized by a strong downward and rearward motion (adduction and retraction) that generates thrust. The cycle concludes with the paddle phase, where the flipper moves ventrally toward the body while maintaining lift before returning to the streamlined position alongside the flank [Figure 7 and Figure 8].

To define a representative baseline stroke for the robotic model, key amplitudes, inflection points, and relative timings were estimated from manual frame-by-frame tracking of sea lions swimming in natural and controlled environments using Kinovea (Version 0.8.0, Kinovea Open-Source Project, Paris, France). These observations were used to construct a parameterized piecewise cubic Hermite interpolating polynomial (PCHIP) model for each actuator degree of freedom: flap, sweep, and twist [26] [Figure 8]. The PCHIP interpolation ensured smooth, monotonic transitions between control points, preventing overshoot in angular motion. This representation was not used for curve fitting but rather as a parametric interpolation framework that reproduces the measured stroke by enforcing monotonic transitions between control points while allowing independent control of amplitude and timing for each joint.

### 2.5. Experiments

#### 2.5.1. Experimental Variables

Experimental trials were conducted to evaluate how variations in stroke kinematics and flow conditions influence the thrust and lift forces generated by the bio-robotic foreflipper. The experiments were divided into three groups: isolated power phase, isolated paddle phase, and full-stroke trials. The recovery phase was excluded from independent testing because its primary function is to minimize drag, not generate propulsive forces.

To account for the mirrored motion of the two biological flippers during natural swimming, only a single foreflipper was modeled and tested. This simplification is justified because the lateral forces produced by the left and right flippers are equal in magnitude and opposite in direction, effectively canceling each other out. Testing one flipper therefore provides a complete representation of the axial thrust and dorsal/ventral lift components without introducing lateral force interactions.

The experimental design manipulated four primary kinematic and flow variables:

Flipper Twist Angle (θ_t_): Varied from 0° (flipper face perpendicular to the flow) to 90° (leading edge aligned with the forward direction) in 15° increments.

Sweep Angle (θ_s_) at Power-Stroke Onset: Set to 70°, 80°, or 90°. Here, 90° represents the flipper positioned orthogonal to the body; 70° represents a slight rearward cant while maintaining full lateral extension [Table 1].

Flow Condition (U): Two flow speeds were tested, 0 m/s (no external flow) and 0.1 m/s (oncoming flow). This binary comparison isolates the influence of ambient flow on flipper-generated thrust and lift.

Phase Overlap (ϕ_ov_): In the full-stroke trials, the temporal overlap between the power and paddle phases was varied. Phase overlap is defined as the percentage of the power phase duration during which the paddle phase occurs simultaneously. The power phase is primarily flap-dominated, whereas the paddle phase is primarily sweep-dominated, enabling controlled initiation of the paddle stroke during the execution of the power stroke. Three overlap percentages were tested: 0% (fully distinct sequential motions), 25% (partial coupling), and 50% (substantial coordination) [Table 1].

The baseline stroke was defined using kinematic parameters derived from the characteristic sea lion propulsive stroke. The flap angle (θ_f_) was set to 45° during the power stroke and 0° during the paddle stroke. The sweep angle (θ_s_) was set to 80° at the end of the recovery phase and maintained at the start of the primary force-producing phases. The baseline phase overlap was 50%. The complete stroke period (*T*) was 2.25 s, corresponding to a frequency of 0.44 Hz (1/*T*). This period is longer than the 1 s stroke observed in biological sea lions. This adjustment was a necessary experimental constraint, as the actuators could not simultaneously overcome the high hydrodynamic loads and achieve the faster biological speeds. While this results in a lower Re than the biological case, this consistent frequency is sufficient for the study’s primary objective: to systematically map the relationship between kinematic changes and the resulting force profiles.

#### 2.5.2. Power Phase Experiments

To isolate the hydrodynamic effects of the flap motion, these trials tested a dynamic flap (θ_f_) while holding the sweep (θ_s_) and twist (θ_t_) joints static. Each trial began with the flipper in a streamlined orientation, transitioning rapidly to the target twist angle (θ_t_, varied from 0° to 90° in 15° increments) within the first 10% of the stroke and holding that angle for the remainder. The starting sweep angle (θ_s_) was also held static at 70°,80°, or 90°. The flap angle (θ_f_) then executed its dynamic PCHIP trajectory (as defined in Section 2.3.1), serving as the primary driver of propulsion during this phase. These kinematics were tested at both flow conditions (0 m/s and 0.1 m/s) and the same period (T = 2.25 s). These combinations produced a total of 42 unique experimental conditions for the power-phase group [Table 1].

#### 2.5.3. Paddle Phase Experiments

To isolate the hydrodynamic effects of the sweep motion, these trials tested a dynamic sweep (θ_s_) while holding the flap (θ_f_) and twist (θ_t_) joints static. Trials began from the end position of the power stroke, with the flap angle (θ_f_) held static at 90°. The twist angle (θ_t_) was also held static at its target value (varied from 0° to 90° in 15° increments) for the entirety of the experiment. The sweep angle (θ_s_) was the only dynamic degree of freedom, executing its PCHIP trajectory from a starting angle of 70°, 80°, or 90° to retract the flipper toward the body. The same nominal frequency and two flow conditions were used, yielding 42 unique experimental conditions for the paddle-phase group [Table 1].

#### 2.5.4. Combined Power and Paddle Experiments

To evaluate the complete propulsive cycle, full-stroke trials combined the dynamic power and paddle phases. These trials were initiated following the reset protocol described in Section 2.5: a streamlined recovery motion to the starting configuration, followed by a 5 s pause to allow tank turbulence to settle. The subsequent propulsive stroke, where all three joints followed their PCHIP trajectories, was parameterized by four primary experimental variables: the power phase twist angle (θ_t_) (varied from 0° to 90° in 15° increments); the starting sweep angle (θ_s_) (set to 70°,80°, or 90°); the phase overlap (ϕ_ov_) (varied at 0%, 25%, or 50%); and the flow condition (U) (tested at 0 m/s and 0.1 m/s). During the initial recovery motion, the twist angle was dynamically set to 90° to align the leading edge with the flow. Based on preliminary testing, the twist angle during the paddle phase was held fixed at 0° (flipper face perpendicular to flow) to maximize thrust. The combination of these parameters resulted in the 126 unique experimental conditions for the full-stroke group [Table 1].

### 2.6. Data Collection and Analysis

Data acquisition and preprocessing were conducted to ensure accurate and consistent force measurements. All force data were collected using a National Instruments NiDAQ (Model USB-6229, TX, USA) at a sampling rate of 250 Hz. The raw signals underwent a two-step filtering process: a median filter (neighborhood size 5) was first applied to remove transient outliers, followed by a low-pass filter (9 Hz cutoff). These filter parameters were selected based on Power Spectral Density (PSD) analysis, which indicated that the majority of signal energy occurred below 9 Hz.

Each experimental condition was repeated multiple times to confirm measurement repeatability. The resulting force traces were highly consistent, and because the standard deviations were negligible relative to the measured forces, uncertainty bands are omitted from the figures for clarity. After filtering, five complete stroke cycles were averaged to obtain a representative force trace for each condition, with the initial five cycles discarded to ensure steady-state behavior. Each experiment was also recorded for five seconds using an underwater camera to verify proper robotic operation and confirm that flipper kinematics matched the programmed trajectories.

The experimental campaign presented in this study consists of 210 distinct conditions (encompassing 1200 individual trials), which are detailed in Section 2.4 and Table 1. These parameters were selected based on additional pilot trials that explored a wider parameter space. This preliminary testing confirmed that while other kinematic variations (e.g., flipper period, alternative sweep/twist angles) produced quantitatively different force magnitudes, they retained the same qualitative trends and waveform characteristics described in this study. The 210-condition dataset presented here therefore provides a comprehensive and representative view of the fundamental relationships between stroke kinematics, flow condition, and propulsive force production.

## 3. Results

### 3.1. Baseline Strokes and Force Metrics

The propulsive forces are first evaluated for a baseline stroke, which serves as a reference for comparison against all kinematic variations. Based on the biological data and the parameters defined in Section 2.4, the baseline stroke is defined by: a starting sweep angle (θ_s_) of 80°, a power phase twist angle (θ_t_) of 45°, a phase overlap (ϕ_ov_) of 50%, and an oncoming flow condition (U) of 0.1 m/s. All strokes are executed with the T = 2.25 s period (0.44 Hz) [Table 2].

**Figure 9:**
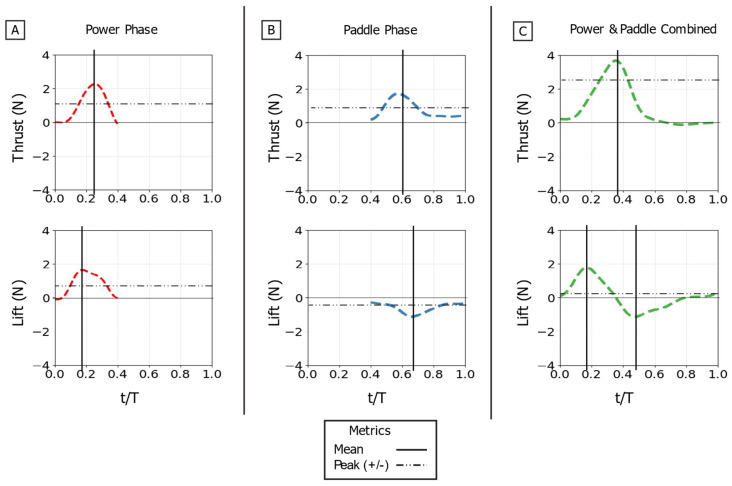
Time-varying thrust and lift traces for the baseline propulsive strokes. Thrust (**top row**) and lift (**bottom row**) are shown for: (**A**) the isolated power phase, (**B**) the isolated paddle phase, and (**C**) the combined power-paddle stroke. The horizontal axis is nondimensional time (t/T), and the vertical axis is force (N). For each trace, the horizontal dotted line indicates the mean force over the phase, and the vertical solid line marks the timing of the peak force.

In the following sections, this paper will first discuss the qualitative shape of the time-varying force traces [Figure 9], as the profile’s features (e.g., peak sharpness, number of peaks, and plateaus) reveal the underlying hydrodynamic mechanisms. To then quantitatively compare conditions, four primary metrics from these traces are extracted:

Peak Force: The maximum positive thrust or lift, or minimum negative lift.

Peak Timing: The time at which the peak force occurs, expressed as a percentage of the complete propulsive phase duration.

Mean Force: The average force generated over the duration of the stroke phase.

Force Vector: The time-varying magnitude and direction of the propulsive force.

#### 3.1.1. Power Stroke Baseline

For the isolated power stroke baseline, the flipper executed its dynamic flap motion while the sweep angle (θ_s_) was held static at 80° and the twist angle (θ_t_) was held static at 45°, under 0.1 m/s flow [Table 2]. The thrust trace begins at zero, rises to a single, sharp peak as the flipper accelerates downward, and then decays as the flipper decelerates. The peak thrust was 2.6 N, occurring at 18% of the full stroke propulsive stroke period. The mean thrust over the phase was 1.1 N. The lift trace shows a similar profile, rising to a positive peak before decaying. The maximum lift force was 1.6 N, occurring slightly earlier than the thrust peak at 14% of the stroke duration. The mean lift was 0.85 N [Figure 9A].

#### 3.1.2. Paddle Stroke Baseline

For the isolated paddle stroke baseline, the flipper executed its dynamic sweep motion retracting from 80°. The flap angle (θ_f_) was held static at 90° and the twist angle (θ_t_) was held static at 0° (face perpendicular to flow), under 0.1 m/s flow [Table 2]. The paddle stroke generates a substantial thrust force. The thrust trace rises rapidly to a peak and is sustained before decaying. The maximum thrust was 1.85 N, occurring at 58% of the period. This generated a mean thrust of 0.85 N. The lift trace begins near zero and then decreases to a strong negative minimum as the flipper sweeps. The minimum lift force was −1.3 N, occurring late in the stroke at 66% of the period, with a mean lift of −0.6 N [Figure 9B].

#### 3.1.3. Combined Power and Paddle Strokes Baseline

Finally, the full baseline stroke was executed with a 45° power phase twist, 80° starting sweep, 50% phase overlap, and 0.1 m/s flow [Table 2]. The force traces show the complex interaction of the two phases. The thrust trace shows one large distinct peak. Due to the 50% overlap, the paddle phase begins while the power phase is still producing thrust, resulting in a maximum combined thrust of 3.8 N at 35% of the total period. The mean thrust for the full cycle was 1.2 N. The lift trace clearly shows this interaction. It rises to a positive peak during the power phase and then rapidly reverses to a negative minimum during the paddle phase. The maximum lift was 1.8 N at 18% of the period, and the minimum lift was −1.6 N at 62% of the period. Because the positive power-phase lift and negative paddle-phase lift are similar in magnitude, they largely cancel each other out over the full cycle, resulting in a mean lift of only 0.15 N [Figure 9C].

### 3.2. Analysis of Isolated Power Stroke Phase

Analysis of the isolated power phase was performed to deconstruct the complex, three-dimensional propulsive stroke into its primary components. This approach allows for the fundamental mechanisms of the dynamic flap motion to be isolated and provides a direct comparison to previous hydrodynamic studies. This characterization of the flap’s fundamental force potential is essential for interpreting the more complex hydrodynamic interactions of the full, combined stroke presented in Section 3.4.

**Figure 10:**
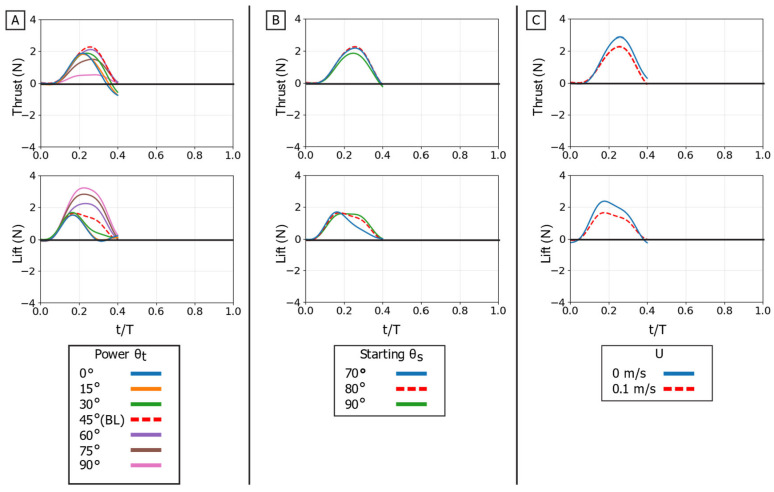
Effect of kinematic variations on isolated power phase forces. Time-varying thrust (**top row**) and lift (**bottom row**) traces are shown for manipulations of the baseline stroke. (**A**) Effect of varying the flipper twist angle (θ_t_) from 0° to 90°. (**B**) Effect of varying the starting sweep angle (θ_s_) from 70° to 90°. (**C**) Effect of the presence of oncoming flow (U). The horizontal axis is nondimensional time (t/T), and the vertical axis is force (N). The baseline (BL) condition is highlighted in each panel for reference.

**Figure 11:**
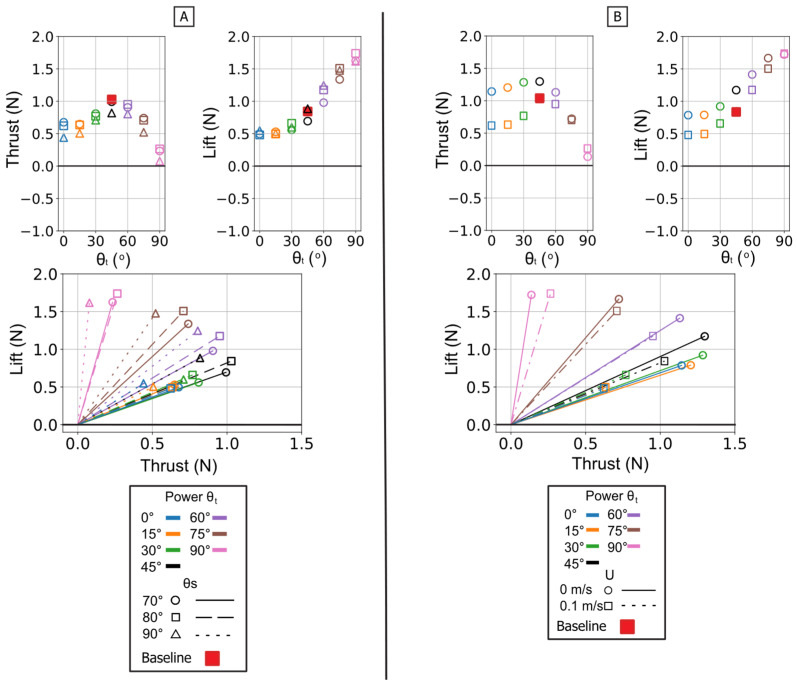
Summary of mean propulsive forces generated during the isolated power phase. Panel (**A**) illustrates the effect of varying the Twist Angle (θ_t_) and Sweep Angle (θ_s_) on mean Thrust (**left**) and mean Lift (**middle**) and Lift vs. Thrust vector (**Bottom**). Panel (**B**) illustrates the effect of varying the Twist Angle (θ_t_) and Flow Speed (U) on mean Thrust (**left**) and mean Lift (**middle**) and Lift vs. Thrust.

The power phase twist angle (θ_t_) was the dominant parameter for partitioning the resultant force vector, creating a clear trade-off between thrust and lift. Mean thrust was non-monotonic, consistently peaking in a broad, robust range centered at θ_t_ = 45° regardless of sweep angle or flow condition. Conversely, mean lift generation was monotonic, increasing from its minimum at 0° to a clear maximum at θ_t_ = 90°. This partitioning effectively rotated the resultant force vector from a thrust-dominant orientation at low angles to a lift-dominant one at high angles [Figure 10 and Figure 11].

These mean-force trends are a direct result of changes in the time-varying force profiles [Figure 10A]. The 45° twist angle produced a thrust trace that was both tall and wide, maximizing the area under the curve (impulse). As the twist decreased from this optimum, the thrust peak became noticeably narrower. As twist increased toward 90°, the peak remained wide, but its magnitude collapsed. The lift traces showed the opposite trend: the 90° twist angle generated the largest and most sustained positive force profile, which monotonically decreased in height and width as the twist angle was reduced.

**Figure 12:**
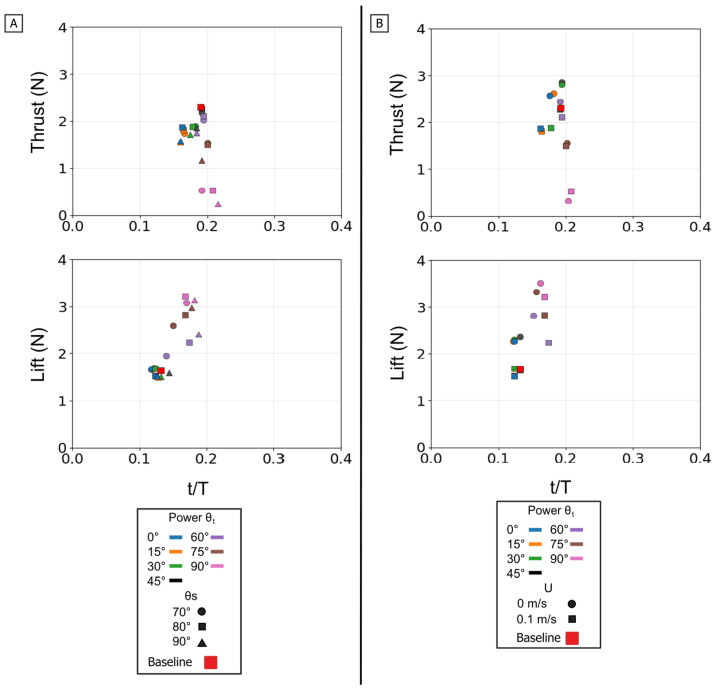
Peak magnitude and peak timing for the isolated power phase. Peak force magnitude (N, *y*-axis) is plotted against nondimensional peak timing (t/T, *x*-axis) for both peak thrust (**top row**) and peak lift (**bottom row**). (**A**) Illustrates the effect of varying the Twist Angle (θ_t_) and Sweep Angle (θ_s_). (**B**) Illustrates the effect of varying the Twist Angle (θ_t_) and Flow Speed (U). The baseline condition is identified by the red square.

The temporal dynamics of the force peaks were also governed by twist angle. For thrust, the 45° twist condition not only produced the highest peak magnitude but also one of the latest-occurring peaks; lower twist angles produced weaker peaks that occurred earlier in the stroke. For lift, both the peak magnitude and the peak timing increased monotonically with θt, shifting from low, early peaks at 0° to high, late peaks at 90° [Figure 12].

The starting sweep angle (θ_s_) was a secondary control for thrust magnitude. A 90° sweep angle consistently produced the lowest mean thrust, regardless of twist. At the critical θ_t_ = 45° thrust optimum, the 80° sweep angle produced the highest mean thrust, outperforming both the 70° and 90° settings [Figure 11A]. While sweep angle had minimal effect on mean lift, it systematically controlled the timing of the lift peak: for any given twist angle, the 70° sweep produced the earliest peak, followed by 80°, with 90° occurring latest [Figure 12A].

The influence of flow speed highlights the relationship between kinematics and force generation. The 0.1 m/s oncoming flow acted as a simple antagonist, systematically reducing the magnitude of both thrust and lift compared to the 0 m/s condition [Figure 11B]. The force profiles for 0.1 m/s and 0 m/s were nearly identical in shape, width, and peak timing, with the 0.1 m/s trace simply reduced in magnitude [Figure 10C]. The optimal thrust angle remained 45°, and the resultant force vectors were reduced in magnitude but not altered in direction [Figure 11B]. This demonstrates that oncoming flow reduces force magnitude without changing the fundamental control map.

In summary, the isolated power stroke functions as a highly adaptable control system. The twist angle (θ_t_) is the dominant control, partitioning the resultant force vector between thrust (optimal at 45°) and lift (optimal at 90°) while also governing the timing of the force peaks. The sweep angle (θ_s_) provides secondary, non-monotonic control of thrust magnitude and systematically adjusts the timing of the lift peak. Finally, oncoming flow acts as a simple antagonist, reducing force magnitude without altering the underlying kinematic relationships.

### 3.3. Analysis of Isolated Paddle Phase

The isolated paddle phase was analyzed to understand its distinct contribution to propulsion. The propulsive forces generated during this phase are controlled by modulating the flipper’s profile area against its direction of motion. This mechanism is fundamentally different from the force partitioning observed in the power phase.

**Figure 13:**
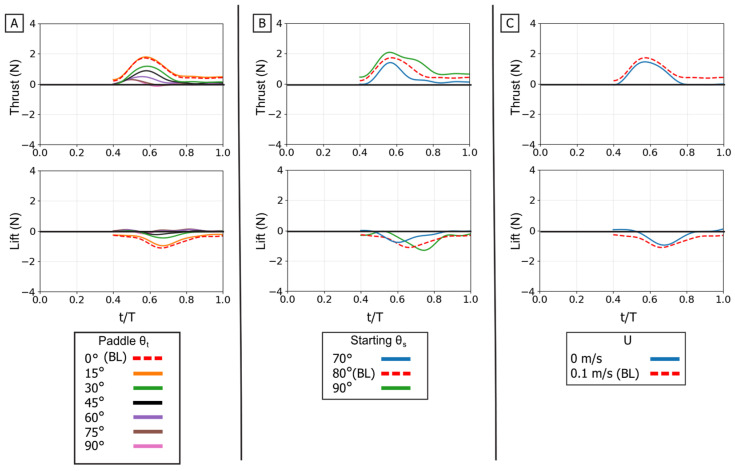
Effect of kinematic variations on isolated paddle phase forces. Time-varying thrust (**top row**) and lift (**bottom row**) traces are shown for manipulations of the baseline stroke. (**A**) Effect of varying the flipper twist angle (θ_t_) from 0° to 90°. (**B**) Effect of varying the starting sweep angle (θ_s_) from 70° to 90°. (**C**) Effect of the presence of oncoming flow (U). The horizontal axis is nondimensional time (t/T), and the vertical axis is force (N). The baseline (BL) condition is highlighted in each panel for reference.

**Figure 14:**
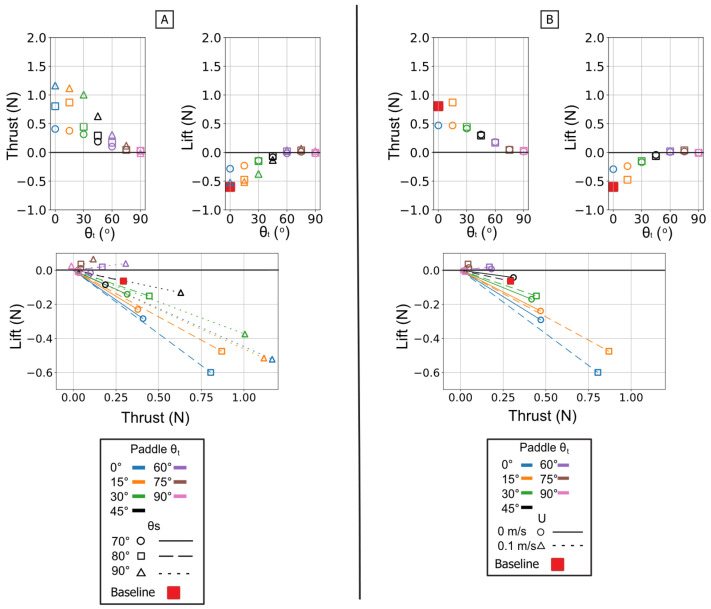
Summary of mean propulsive forces generated during the isolated paddle phase. Panel (**A**) illustrates the effect of varying the Twist Angle (θ_t_) and Sweep Angle (θ_s_) on mean Thrust (**left**) and mean Lift (**middle**) and Lift vs. Thrust vector (**Bottom**) Panel (**B**) illustrates the effect of varying the Twist Angle (θ_t_) and Flow Speed (U) on mean Thrust (**left**) and mean Lift (**middle**) and Lift vs. Thrust.

The twist angle (θ_t_) was the dominant parameter for controlling the overall force magnitude. In contrast to the power phase where twist partitioned forces, here it functioned as the primary control for force magnitude by modulating the flipper’s effective surface area. Both mean thrust and mean negative lift were maximal at θ_t_ = 0° (flipper face perpendicular to motion). As the twist angle increased, feathering the flipper, both mean force components monotonically decreased, approaching zero at θ_t_ = 90°. All resultant force vectors resided in the fourth quadrant (positive thrust, negative lift), and their magnitude was largest at low twist angles, shrinking toward the origin as twist increased [Figure 13 and Figure 14].

**Figure 15:**
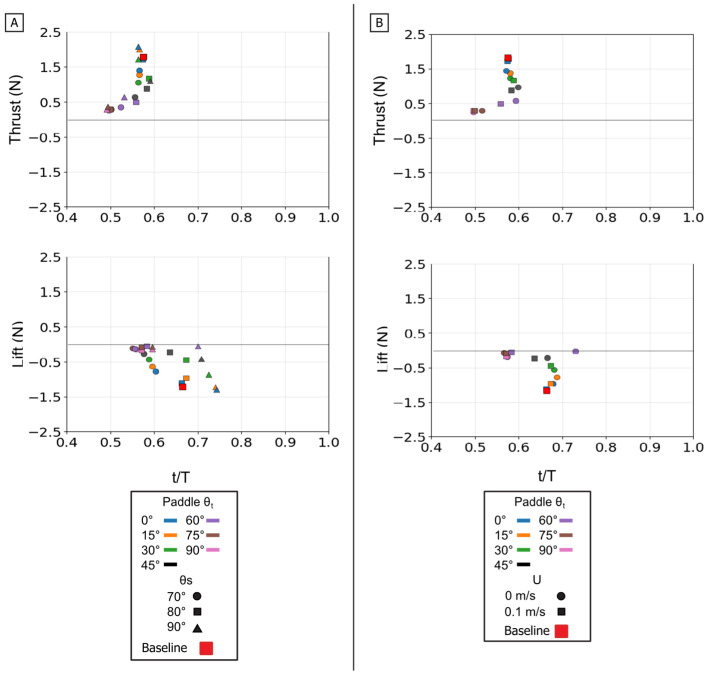
Peak magnitude and peak timing for the isolated paddle phase. Peak force magnitude (N, *y*-axis) is plotted against nondimensional peak timing (t/T, *x*-axis) for both peak thrust (**top row**) and peak lift (**bottom row**). (**A**) Illustrates the effect of varying the Twist Angle (θ_t_) and Sweep Angle (θ_s_). (**B**) Illustrates the effect of varying the Twist Angle (θ_t_) and Flow Speed (U). The baseline condition is identified by the red square.

This geometric control mechanism is a direct result of the changes in the force profiles. The 0° twist angle produced the tallest and widest peaks for both thrust and negative lift, maximizing the impulse. As twist increased, these force profiles rapidly collapsed toward the zero-force line [Figure 13A]. The temporal dynamics were also affected: as twist angle increased, the force peaks occurred progressively earlier in the stroke, shifting from approximately 60–70% of the period duration at 0° to 45–55% at 90° [Figure 15].

The starting sweep angle (θ_s_) was a secondary control that systematically modulated thrust magnitude. A clear monotonic trend was observed, where the 90° sweep angle consistently produced the highest mean thrust, followed by 80°, and 70° [Figure 14A]. This corresponds to the force traces, where the 90° sweep produced a higher and broader thrust peak than the 70° sweep [Figure 13B]. Sweep angle had a minimal and non-systematic effect on lift magnitude.

Finally, a key finding of this phase is its relative insensitivity to oncoming flow (U). Unlike the power phase, where flow was a clear antagonist, the paddle stroke’s performance was nearly identical with or without flow. The force traces for 0 m/s and 0.1 m/s are almost indistinguishable in shape, timing, and magnitude. This observation is strongly supported by the mean and peak force data, which show no significant, systematic difference between the 0 m/s and 0.1 m/s conditions. This flow independence is consistent with the dominance of pressure drag in the bluff-body orientation (0° twist); in this regime, the flow separation is determined primarily by the flipper’s high-velocity motion relative to the fluid, rendering the forces robust to the weak ambient flow [Figure 13C, Figure 14B and Figure 15B].

### 3.4. Analysis of Combined Power and Paddle Phase

The full propulsive stroke analysis reveals the complex interaction between the force-partitioning mechanism of the power phase and the geometrically driven thrust of the paddle phase. This section investigates how the combined kinematics create a tunable and powerful propulsive system. To isolate the primary control variables, the paddle phase twist angle (θ_t_) was held constant at 0°, its optimal thrust setting as determined in Section 3.3. Therefore, all manipulations of twist angle (θ_t_) discussed in this section refer exclusively to the power phase twist angle.

**Figure 16:**
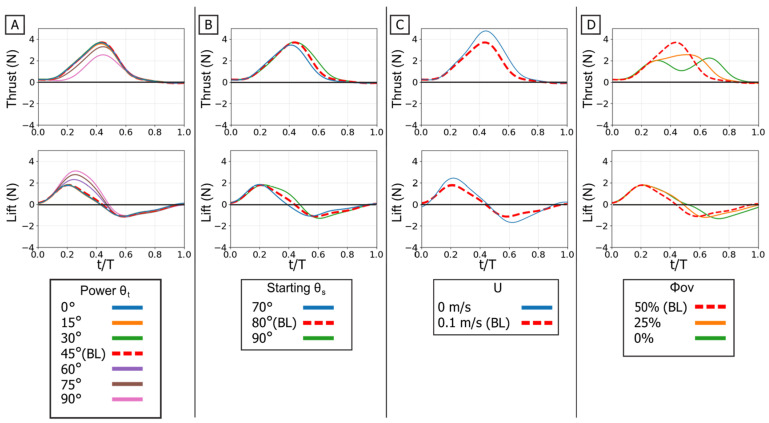
Effect of kinematic variations on full-stroke forces. Time-varying thrust (**top row**) and lift (**bottom row**) traces are shown. (**A**) Effect of varying the flipper twist angle (θ_t_). (**B**) Effect of varying the starting sweep angle (θ_s_). (**C**) Effect of the presence of oncoming flow (U). (**D**) Effect of varying the phase overlap (ϕ_ov_). The horizontal axis is nondimensional time (t/T), and the vertical axis is force (N). The baseline (BL) condition is highlighted for reference.

**Figure 17:**
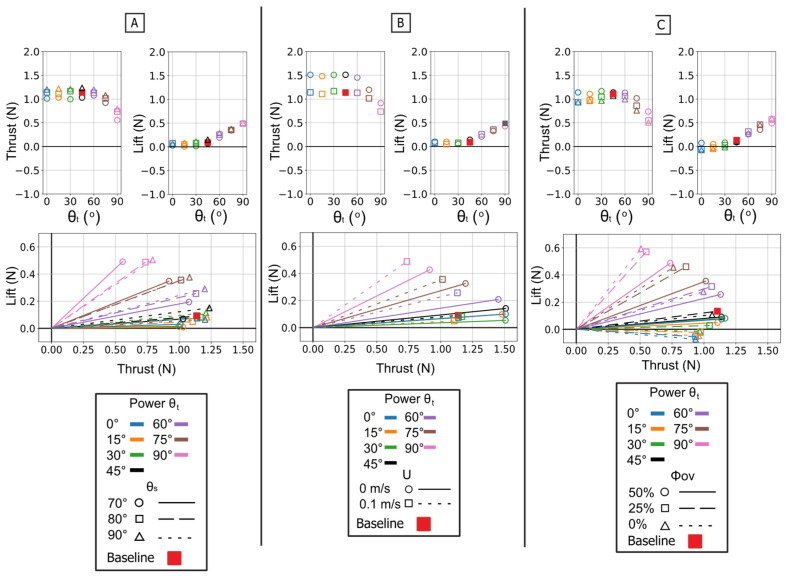
Summary of mean propulsive forces generated during the full stroke. Panel (**A**) illustrates the effect of varying the Twist Angle (θ_t_) and Sweep Angle (θ_s_) on mean Thrust (**top left**) and mean Lift (**top right**). Panel (**B**) illustrates the effect of varying the Twist Angle (θ_t_) and Flow Speed (U). Panel (**C**) illustrates the effect of varying the Twist Angle (θt, colors) and Phase Overlap (ϕ_ov_). The plots in the bottom row show the corresponding force vectors (Lift vs. Thrust) for each set of conditions. The baseline condition is identified by the red square.

**Figure 18:**
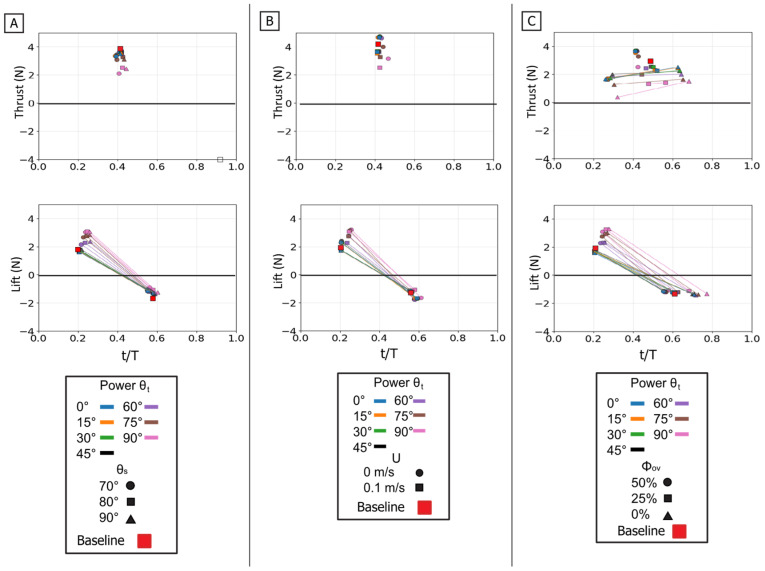
Peak magnitude and peak timing for the full stroke. Peak force magnitude (N, *y*-axis) is plotted against nondimensional peak timing (t/T, *x*-axis) for both peak thrust (**top row**) and peak lift (**bottom row**). (**A**) Illustrates the effect of varying the Twist Angle (θ_t_) and Sweep Angle (θ_s_). (**B**) Illustrates the effect of varying the Twist Angle (θ_t_) and Flow Speed (U). (**C**) Illustrates the effect of varying the Twist Angle (θ_t_) and Phase Overlap (ϕ_ov_). For each condition (color), thin lines connect the multiple force peaks (e.g., positive power-phase lift and negative paddle-phase lift) that occur within that single stroke cycle. The baseline condition is identified by the red square.

Temporal phase overlap (ϕ_ov_) was found to be a primary mechanism for thrust augmentation. Increasing the temporal overlap between the power and paddle phases resulted in the constructive combination of their individual thrust peaks. At 0% overlap, the stroke produced two distinct, smaller thrust peaks, corresponding to each phase. As overlap increased to 50%, these peaks merged into a single, substantially larger thrust pulse [Figure 16D]. This merging of impulse directly resulted in higher mean thrust, which was lowest at 0% overlap and highest at 50% [Figure 17C]. The increase in peak and mean force with phase overlap can be described as constructive interference of the impulse events. As the overlap increases, the acceleration-driven thrust of the paddle phase initiates before the power phase thrust has decayed. This temporal superposition merges the two distinct force peaks into a single, amplified impulse, increasing the total mean thrust. This temporal-to-magnitude coupling is further confirmed by the peak force data, which shows the two distinct peak clusters at 0% overlap coalescing into one high-magnitude cluster at 50% [Figure 18C].

The power phase twist angle (θ_t_) functions as the primary control for partitioning the net propulsive force vector, creating a distinct trade-off between straight-line thrust and vertical lift. Mean thrust was maximized and relatively flat between θ_t_ = 0° and 45°. In this same range, the net mean lift was near zero because the positive lift of the power phase and the negative lift of the paddle phase effectively cancelled each other out. However, as θ_t_ increased above 45°, this force symmetry was broken. The positive power-phase lift peak was dramatically amplified, while the negative paddle-phase peak remained relatively unchanged [Figure 16A]. This unbalancing of the vertical forces caused the observed sharp decline in mean thrust and a monotonic increase in net positive mean lift. The resultant force vectors confirm this steering function: at low twist angles, the vectors were aligned almost purely with the thrust axis, but as twist increased, they rotated progressively upward toward the lift axis [Figure 17].

The starting sweep angle (θ_s_) and oncoming flow speed (U) act as secondary modulators of the combined stroke. Sweep angle provided fine-tuning of thrust magnitude; the 70° sweep angle consistently produced the lowest mean thrust across all twist angles, while the 80° and 90° sweeps yielded similar, higher thrust values [Figure 17A]. The net influence of oncoming flow was a superposition of its effects on each isolated phase. The 0.1 m/s flow reduced the magnitude of the initial power-phase peak but had no discernible effect on the subsequent paddle-phase peak [Figure 16C]. Because the power phase is antagonistic to the flow, its diminished contribution resulted in a net mean thrust that was systematically lower in 0.1 m/s flow than in 0 m/s flow [Figure 17B].

In summary, the full propulsive stroke operates as a highly adaptable and integrated system. Phase overlap (ϕ_ov_) is the dominant control for thrust magnitude, merging the two stroke components via constructive hydrodynamic interaction. The power phase twist angle (θ_t_) serves as the primary steering control for force direction, partitioning the net output between pure thrust (optimal at 0–45°) and a combined thrust–lift vector (at θ_t_ > 45∘). This robust control map provides a clear framework for bio-inspired vehicles to balance high-efficiency, straight-line propulsion with agile, three-dimensional maneuvering.

## 4. Discussion

This study characterized the time-varying thrust and lift produced by a bio-robotic sea lion foreflipper to identify the kinematic mechanisms that control force generation and reorientation. The results provide a systematic, time-resolved mapping between flipper kinematics and the instantaneous propulsive force vector. The findings show that within the tested inertial flow regime (Re ≈ 10^4^–10^5^), the foreflipper operates as a hybrid propulsion system, producing powerful thrust and lift forces in a single stroke, and its forces can be effectively controlled by geometric and temporal kinematics. This study provides a foundational map for these control mechanisms.

A central finding of this work is the robust, high-angle thrust optimum of the power phase. The results show mean thrust is maximized in a broad range between 0° and 45° (peaking at 45° in the isolated stroke, as shown in Figure 11), and then drops off sharply at higher angles. This is a critical result because many propulsive-foil analyses are interpreted through a lift-based framework, which would predict an optimal thrust at a low, attached-flow angle of attack (e.g., AoA ≈ 10–15°) [23,25]. In contrast, the robust thrust optimum observed near 45° is consistent with a drag-based or bluff-body propulsive mechanism rather than attached-flow lift. At this high angle of attack, the flipper effectively operates in a post-stall regime, where the large pressure differential across the surface generates a resultant force vector roughly normal to the chord. The 45° orientation maximizes the projection of this normal force along the thrust axis. While the experiments, which did not measure the flow field, cannot definitively rule out lift-based effects, the force data—particularly this high-angle, robust optimum and its insensitivity to oncoming flow—are highly consistent with a system dominated by geometric momentum redirection and impulse timing.

This emphasis on geometric mechanisms is also a direct consequence of the model’s simplified design. The biological flipper possesses a high aspect ratio (4.1–7.9), spanwise flexibility, and chordwise taper, all of which are features that can enhance circulation-based lift. The model, by design, employed a low aspect ratio (2.7), rectangular planform, and a single compliant hinge. These design choices deliberately simplify the hydrodynamic system, resulting in a device that functions as a simplified “paddle,” emphasizing pressure-driven thrust. This makes the model highly appropriate for isolating and studying the foundational geometric basis of propulsion, though not for replicating the full, flexible, high-aspect-ratio performance of the biological foreflipper. The findings should therefore be viewed as identifying the foundational geometric mechanism that likely acts in concert with lift-based effects in the real animal.

The experiments also demonstrate how geometric and temporal control of kinematics can generate a predictable range of thrust and lift vectors. The power phase twist angle governs the direction of the resultant force vector, the paddle phase twist sets thrust magnitude by adjusting the flipper’s presented area, and temporal phase overlap regulates the overall impulse by merging sequential force peaks. Together, these parameters form a controllable framework for agile propulsion. This map is not intended to replicate sea lion performance directly but to show how simple, geometry-based rules can produce reliable force reorientation in underwater robots operating at similar Reynolds numbers.

The dual-phase structure of the stroke highlights a hybrid strategy for combining versatility and robustness. The power phase acts as a steering stroke that reorients the propulsive vector, while the paddle phase provides strong, flow-insensitive thrust. This division of labor allows propulsion that is both adaptable and consistent, a useful design principle for robotic vehicles. This behavior establishes the baseline control logic for the geometric component of the stroke, upon which more complex, lift-augmented mechanisms may be layered in the biological system.

Several limitations define the scope of these findings. The simplified geometry and slower stroke frequency (T = 2.25 s), which lowers the flipper’s self-induced velocities, are the primary constraints. The simplified model, while useful for isolating mechanisms, means these findings cannot be used to describe the full dynamics of the flexible, high-aspect-ratio biological flipper. The study provides a map of the geometric component; it does not quantify the lift-based component. Additionally, the experimental Reynolds numbers (3.3 × 10^4^ to 6.6 × 10^4^) reside in the inertial regime but remain below peak biological values. While the geometric momentum redirection described here is governed by inertial forces and expected to persist, the relative contribution of circulation-based lift likely increases at full scale. At higher Reynolds numbers, reduced boundary layer thickness may sustain attached flow over a wider range of angles. Therefore, the present control map serves as the geometric baseline upon which lift dynamics are likely superimposed in the biological system. Future work should explore how this geometric baseline couples with lift-based dynamics. Increasing stroke frequency, incorporating distributed flexibility, and testing with full planform geometry will help determine how biological flippers integrate these two propulsive mechanisms across scales. Finally, while this study systematically maps the integrated propulsive forces, it does not employ flow visualization techniques (e.g., PIV) to resolve the specific vortex structures associated with these forces. Future work will aim to correlate these force measurements with flow field topology to further validate the geometric mechanisms proposed here.

In summary, the present results identify geometric momentum redirection and impulse timing as dominant propulsive mechanisms in this simplified, low-aspect-ratio robotic model. These mechanisms describe a fundamental geometric control of thrust and lift that likely provides the structural foundation upon which circulation-based lift develops at biological scale. The study thus complements, rather than contradicts, previous research that has focused on lift-based interpretations by revealing and systematically mapping the foundational geometric origins of the stroke.

## 5. Conclusions

This study experimentally quantified the time-varying thrust and lift generated by a bio-robotic sea lion foreflipper to identify how kinematic inputs—specifically twist, sweep, and phase overlap—govern instantaneous propulsive forces. The analysis revealed that the foreflipper stroke operates as a highly adaptable, hybrid propulsion system composed of two hydrodynamically distinct components. The power phase functions as a versatile force-vectoring stroke, where twist angle partitions the resultant force between thrust, which exhibits a broad, robust optimum peaking near 45°, and lift, which increases toward 90°. This high-angle, robust optimum remained consistent across flow conditions, indicating a geometric control mechanism rather than sensitivity to oncoming flow. The paddle phase acts as a robust, geometrically driven thrust generator, where force is maximized at 0° twist, when the flipper’s face is broadest to the direction of motion and remains insensitive to external flow.

When combined, the temporal-phase overlap merges the individual thrust peaks into a single, amplified impulse, while the power-phase twist angle governs the overall direction of the resultant force vector. These findings describe a coherent control framework in which the twist governs the direction, the presented area governs the magnitude, and the overlap governs the total impulse.

This entire control map, particularly the high-angle, robust optimum, is highly consistent with propulsion dominated by geometric momentum redirection and impulse timing, in contrast to the low-angle optimums typical of lift-based foils. These findings, enabled by a simplified, low-aspect-ratio model designed to isolate these effects, define the foundational geometric basis of the stroke. This study thus complements, rather than contradicts, research focused on lift-based interpretations by systematically mapping a mechanism that likely acts in concert with circulation-based effects in the biological animal. This framework provides a practical and scalable foundation for the design and control of agile, bio-inspired underwater vehicles.

## Figures and Tables

**Table 1:** Independent Variables for Each Phase of Experimentation.

Experimental Parameters	Power Stroke	Paddle Stroke	Combined Power and Paddle Stroke
Power θ_t_ (°)	0, 15, 30, 45, 60, 75, 90	—	0, 15, 30, 45, 60, 75, 90
Paddle θ_t_ (°)	—	0, 15, 30, 45, 60, 75, 90	0
Starting θ_s_ (°)	70, 80, 90	70, 80, 90	70, 80, 90
Period (s)	2.25	2.25	2.25
U (m/s)	0, 0.1	0, 0.1	0, 0.1
ϕ_ov_ (%)	—	—	0, 25, 50
Total Experimental Conditions:	42	42	126

**Table 2:** Baseline Stroke Settings by Phase.

Baseline Stroke Settings	Power θ_t_ (◦)	Paddle θ_t_ (◦)	Starting θ_s_ (◦)	ϕ_ov_ (%)	U (m/s)	Period (s)
Power Phase	45	—	80	—	0.1	2.25
Paddle Phase	—	0	80	—	0.1	2.25
Combined Power and Paddle	45	0	80	50	0.1	2.25

## Data Availability

The data generated during the study are available from the corresponding author on reasonable request.
